# Attracting Child Psychiatrists to a Televideo Consultation Service: The TeleLink Experience

**DOI:** 10.1155/2013/146858

**Published:** 2013-06-20

**Authors:** Tiziana Volpe, Katherine M. Boydell, Antonio Pignatiello

**Affiliations:** ^1^Community Health Systems Resource Group, The Hospital for Sick Children, 555 University Avenue, Toronto, ON, Canada M5G 1X8; ^2^Child Health Evaluative Sciences, Research Institute, The Hospital for Sick Children, 555 University Avenue, Toronto, ON, Canada M5G 1X8; ^3^Dalla Lana School of Public Health, University of Toronto, 155 College Street, 6th Floor, Toronto, ON, Canada M5T 3M7; ^4^Department of Psychiatry, Faculty of Medicine, University of Toronto, 250 College Street, 8th Floor, Toronto, ON, Canada M5T 1R8; ^5^Department of Telepsychiatry, TeleLink Mental Health Program, The Hospital for Sick Children, 555 University Avenue, Toronto, ON, Canada M5G 1X8

## Abstract

*Objective.* Identify aspects of psychiatry work that are rewarding, as well as those that are challenging, from the perspective of psychiatrists and residents participating in televideo consultation services. *Method*. A web-based survey was distributed to psychiatrists within the Division of Child Psychiatry at the University of Toronto. Also, semistructured interviews were conducted with six child psychiatrists providing services to a telepsychiatry program. Finally, a focus group interview was held with four psychiatry residents. *Results*. Child psychiatrists are very comfortable conducting assessments via televideo. Factors identified as being important in the decision to participate in telepsychiatry include assisting underserved communities, supportive administrative staff, enhanced rural provider capacity, financial incentives, and convenience. The study's qualitative phase identified four themes in the decision to participate in telepsychiatry: (1) organizational, (2) shared values, (3) innovation, and (4) the consultation model. *Conclusion*. The success of televideo consultation programs in attracting child psychiatrists to provide consultation services to underresourced communities makes an important contribution to psychiatric workforce shortages. Understanding what aspects of telepsychiatry are most appreciated by consulting psychiatrists and residents offers useful strategies to telepsychiatry administrators and medical school educators seeking to attract, train, and retain psychiatry practitioners.

## 1. Background

There is an emerging concern about the shortage of child psychiatrists in Canada and elsewhere [1, 2], a problem that is expected to worsen in the coming years. As availability of psychiatric expertise diminishes, access to timely and appropriate specialty mental health care for children and youth is endangered. In Ontario, Canada, the problem of access is particularly problematic in rural communities and complicates provision of mental health services [3]. The ratio of child psychiatrists to children with mental health needs is approximately 1 : 6,148 [4]. However, only 2% of child psychiatrists practice primarily in areas with populations of less than 20,000, though approximately 18% of the population lives in rural areas [4]. The shortage of resources and support services in rural communities means that children requiring urgent care often are frequently placed in residential care outside of their community [5].

Live interactive videoconferencing technology offers an innovative opportunity to address the shortage of child psychiatrists in Canada [6]. The use of interactive video technology is endorsed by The Canadian Academy of Child Psychiatry [2]. Also, the Canadian Standing Senate Committee on Social Affairs, Science, and Technology [7] recommends that telepsychiatry be used in rural and remote communities for consultations, education, and training of mental health practitioners.

## 2. The TeleLink Mental Health Program

In 1997, The Hospital for Sick Children (SickKids), in Toronto, Ontario, undertook a pilot project to provide mental health support to primary care settings through videoconferencing. Fully operational in 2000, the program evolved to become the TeleLink Mental Health Program. The program aims to enhance the knowledge, skill set, and confidence of children's mental health practitioners using videoconferencing and other technologies by providing timely, equitable access to bilingual (English and French) specialist services. 

Videoconferencing between recipient “far” sites and TeleLink “hub” site occurs by way of Internet Protocol (IP), (maximum bandwidth 384 Kbits/second). Two or more sites can be connected simultaneously, and videos, powerpoint, and scanned documents may be transmitted. The hub site is equipped with five stationary studios, each utilizing a 50 inch plasma or LCD monitor, and one mobile unit containing a 17 inch LCD monitor. Both hub and far sites can be viewed simultaneously, and all cameras have local and remote pan-tilt-zoom control. Polycom or Tandberg videoconferencing units enable the data transmission. Infrastructure support is provided by core staff at the hub and a designated telepsychiatry coordinator at far sites.

Referral routes are multiple: 15 primary children's mental health agencies in rural Ontario (including satellite locations); three community hospitals; one youth detention centre; one community youth justice diversion program; and community physicians. Models of service delivery include clinical consultation and/or short-term follow-up, professional to professional consultation, shared care, program consultation, and education and training. As a consultative program, responsibility for the case remains primarily with the referring agency or physician, with the consulting psychiatrist neither accepting ongoing responsibility for primary care, nor prescribing medication. Referring clinicians complete a mental health assessment before the consultation. Supporting documentation such as past psychological/psychoeducational evaluations, school reports, and screening tools, is also requested. It is highly encouraged that the client's primary clinician/case manager be present during the session; other participants may include parents/caregivers and others involved in the child's care.

Since its inception, the TeleLink Mental Health Program has successfully recruited a stable core of consulting child psychiatrists. Twenty-three child psychiatrists within the Division of Child and Adolescent Psychiatry at the University of Toronto provide the bulk of services through a regular weekly or monthly roster, as determined by the consultant's interest, availability, and commitment. An additional 16 faculty members are called on regularly for specific consultations and educational sessions, depending on their expertise and availability. Every faculty member must have active privileges at The Hospital for Sick Children.

The recruitment of child psychiatrists into the TeleLink Mental Health Program has grown since its inception. However, little is known about the factors that may influence the decision of residents and child psychiatrists to work with rural and remote communities via televideo. In a child and youth mental health system that is fragmented, underfunded, and with a critical shortage of specialists in rural areas, it is important to identify the factors that could enhance recruitment into telepsychiatry and contribute to the expansion of telepsychiatry services to rural and remote communities. This study identifies aspects of psychiatry work that are most rewarding, as well as those that are challenging, from the perspective of psychiatrists and residents participating in televideo consultation services. 

## 3. Methods

To enhance our understanding of psychiatrists' decisions to participate in the provision of services via televideo, this study used a mixed method design, combining quantitative and qualitative methods. For the quantitative component, a web-based survey was developed using SurveyGizmo online software. For the qualitative phase, in-depth interviews and a focus group were conducted following Denzin's [8] interpretive interactionist framework. 

### 3.1. Data Collection

The survey was distributed via e-mail to all child psychiatrists (*n* = 80) within the Division of Child and Adolescent Psychiatry at the University of Toronto. The distribution sample was based on the fact that the TeleLink Program is well promoted within the division and all faculty members are eligible to participate in the Program. The survey included questions related to their experiences with the TeleLink Program (see Tables 1–5). Twenty-six responses (32.5%) were received, a response rate exceeding expectations for web-based surveys (27.4%) [9, 10]. 

The survey used was developed specifically for this study. It was created by the authors based on their collective experience in the field [6], literature review, and consultation with key stakeholders. Its design was informed by a social ecological framework that highlights the need to address phenomena from the interaction among many factors at the individual, relationship, community, and societal level [11].

Focus groups and semistructured individual interviews were conducted to provide a more detailed examination of the survey questions. Invitations to participate in a focus group were sent to all division child and adolescent psychiatrists (*n* = 80) and all psychiatry residents (*n* = 26) located at The Hospital for Sick Children at the time of data collection. Despite multiple attempts to engage focus group participants, our efforts to recruit psychiatrists were unsuccessful. As such, one focus group was held with four psychiatry residents. For the individual interviews, eight individuals involved or familiar with pediatric telepsychiatry services were approached, both in Ontario and internationally. Six individuals agreed to participate; four providing services to the TeleLink Program in Ontario and two providing services to programs outside the country, one in the United States and one in Australia. This sampling strategy, focused on “information-rich cases” [12], was selected as most productive in helping us understand the factors that influence the decision to participate in a telepsychiatry program and those that may be unique to the TeleLink Program. Interviews lasted approximately 40–60 minutes and were conducted either face-to-face or by telephone. A semistructured interview guide directed the interviews and focus group discussion (see Table 6). As with the survey, the interview guide was informed by the authors' previous work, the literature, and consultation with key informants. 

### 3.2. Data Analysis

Survey response data were analyzed using simple frequency statistics and graphic representations. An open-ended text report assisted in the analysis of multiple choice questions as well as verbatim responses. Qualitative interviews were audiotaped and transcribed. The interview and field note transcripts were analyzed using an interpretive interactionist framework [8]. The first step of the analytic process is bracketing, followed by construction and contextualization of findings. Bracketing involves isolating the essential elements under investigation, a process commonly known as coding. The process of construction classifies, orders, and reassembles the phenomenon back into a coherent whole. Finally, with contextualization, greater meaning is sought across individual experiences. 

## 4. Results

### 4.1. Part 1: Online Survey

The 26 survey respondents consisted of 14 (53.8%) males and 12 females (46.2%), ranging in age from under 34 to over 65 and averaging 47.1 years. There was a range of experience in working as a child psychiatrist, from 1 to 47 years, with a median of 21 years. Over 80% of respondents (*n* = 21) indicated that more than 50% of their practice was dedicated to working with children and adolescents. The type of services provided included outpatient (88%), consultation (80%), private practice (40%), in-patient (36%), and others (32%).

Survey respondents were all familiar with the TeleLink Program but diverse in terms of their experience with the Program. While more than one third indicated that they provide frequent consultation services of more than 12 per year (34.6%), there were respondents who had never provided services (23.1%) (Table 1). The majority (68%) indicated that telepsychiatry is an important innovation that allows access to much needed resources in underserviced communities and that it is important for psychiatric trainees to develop expertise in providing consultation. To this end, 68% of respondents recommend that the University's curriculum committee make telepsychiatry mandatory for subspecialty training. Those who resist mandatory telepsychiatry training believe that people are more likely to choose it if it is not forced upon them. 

Working with TeleLink technology was not identified as a significant barrier. As reported in Tables 2 and 3, respondents indicate that they are moderately (20%) to very comfortable (60%) conducting assessments via televideo (with 72% selecting 7 or above on a 10-point Likert scale), as well as with operating the video equipment and associated technology (40% and 56%, resp.). 

At least 50% of respondents identify a number of factors as being very important or important in their decision to provide psychiatric services via televideo, including the ability to provide services to underserviced communities, the support they receive from TeleLink administrative staff, contributing to the enhancement of rural provider capacity, and maintaining ongoing relationships with rural providers, financial incentives, and convenience (Table 4). The novelty of the technology, privileges associated with a hospital appointment, and the opportunity to be a part of a larger group of professionals were of relatively lower importance. The management of the Program, supporting TeleLink's mission, working within a consultation model, trying out telepsychiatry on a trial basis and diversification of income sources were not identified at all as influencing the decision to become involved. 

Survey respondents were asked to indicate the extent to which they agreed with a series of statements about the delivery of psychiatric services via televideo (Table 5). The majority (76%) agreed/strongly agreed that the technology was secure and confidential although about one-third (33.3%) expressed concerns about safety and liability issues. More than half (56%) desired more feedback about the helpfulness of the consultation. There was some need expressed for more knowledge about the particular culture of the community (20%) and for more awareness of the specific resources available in rural communities (52%). 

Respondents were asked to identify the circumstances that would allow them to provide more hours to the TeleLink Program. Responses included flexibility to see the child on more than one occasion, increased time for teaching and training, and review of documents, opportunity to work more closely with community physicians and to have their follow-up and support. 

### 4.2. Part 2: Qualitative Interviews and Focus Group

For the qualitative phase of the study, participants were asked to discuss their involvement in a telepsychiatry program and the factors that may encourage or deter their participation. Four main factors were identified: (1) organizational; (2) shared values; (3) innovation; and (4) the consultation model.

*(1)  Organizational Factors*. An important component in recruiting and retaining consulting psychiatrists is the support provided by the program's executive and administrative staff. This result is supported in the survey data, with 96% of respondents identifying staff support as very important or important in their decision to participate in a telepsychiatry Program. Interview participants identified “*friendly staff, welcoming environment and comfortable set-up*” as influencing factors. Consultants with the TeleLink Program cited “*effective leadership*” by the TeleLink medical director, and his visibility within the department of psychiatry as important aspects of participation. Psychiatrists noted that strong administrative and technical support ensures worry-free consultation sessions. 

As revealed in the survey data, the TeleLink Program's ability to provide a flexible work environment and willingness to adapt to consultant needs emerged as a deciding factor. In the qualitative interviews, further exploration revealed that prospective consultants, uncertain about participating, appreciated the offer of a trial period without obligation. Further tailoring included flexibility regarding the number of hours worked per week or month and the type of cases offered. Participants acknowledged that it “*takes time*” to become comfortable with televideo, and the option to try it out on a trial basis is a facilitator. For one international participant, the increasing demands on pediatric teaching hospitals and the psychiatry department make participation in telepsychiatry difficult to negotiate. 

While most consultants find participating in the TeleLink Program convenient, those located at a distance from the Program's head office find it inconvenient and costly given commuting times and parking costs. One international participant notes that her institution schedules several televideo consultations in one day, similar to a half-day clinic, to make participation more attractive to consulting psychiatrists. 

*(2)  Shared Values*. In keeping with survey respondents, interviewees indicate that a shared sense of purpose and belief in the mission of telepsychiatry is an important aspect of participation. Involvement in telepsychiatry allows consultants the opportunity to improve access to mental health services in rural and remote communities and support local health practitioners. Also in keeping with survey data, interview participants value the opportunity to enhance the knowledge and skills of rural clinicians, recognizing it as “*an innovative way to extend your skills beyond your local practice, and serve great benefits to rural communities*.” Psychiatrists providing regular service to particular sites develop a strong connection and beneficial relationship with the rural clinicians and wider community.

The sense of contributing to a shared mission and a collective pride in program accomplishments is attributed to the staff retention rates. The fact that a program is well supported within the psychiatry department and showcased nationally and internationally enhances its profile. One consultant with TeleLink stated that the “*synchronicity of purpose*” between the Program and its consultants is the vital ingredient in recruiting dedicated child and adolescent psychiatrists. He goes on to say that “*retention is not just about money*”, but rather creating “*an environment where an individual feels comfortable, recognized, contributory to the mission*.” 

*(3)  Innovation*. The role of innovation and technological advances in bringing scarce psychiatric resources to underserved communities emerged as a key factor to both the quantitative and qualitative participants. While survey data suggests that only 12% of respondents identify “novelty” of the technology as important or very important in their decision to provide services via televideo, 96% reported the opportunity to provide services to rural communities as important/very important. In our individual interviews, participants explore this theme further, acknowledging the increasing use of  “*electronic connections”  *such as Skype and Facebook in modern culture, the appeal this has for younger generations, and its potential role in attracting medical students to the field of psychiatry. Psychiatry residents in the focus group session concur, describing the delivery of mental health services via televideo as a creative way to deliver a scarce resource to communities in need. 

In both interview and focus group data, the TeleLink Program is credited with offering psychiatric residents unique training opportunities (each of the core child residents at the University of Toronto must participate in two telepsychiatry consultations, during their six-month rotation). In particular, it is seen as addressing important learning gaps, including how to do a consultation, work collaboratively, and use televideo technology effectively. According to the psychiatric residents, participating in telepsychiatry sessions is rewarding, particularly if the consulting psychiatrist provides opportunities to participate on camera and ask questions. Being outside of the camera's view was difficult, awkward, and not as rewarding. According to one participant, this positive experience may be “*one of the little things that tip them towards child psychiatry*.”

Both interview and survey data reveal a high level of comfort with technology among consultants, with many stating little or no differences between video and face-to-face consultations. Others identify the immediacy of the service as important, as well as the ability to follow-up with patients via video. While participants noted that the technology does not interfere with performing a mental health assessment, some difficulties were identified such as poor picture quality, audio lag, uncertainty in operating the equipment, and unstable internet connections in remote communities. Also, one participant noted that room setup and camera placement at the far sites are not always ideal. Despite these difficulties, one participant states that the advantages of telepsychiatry “*far outweigh these small disadvantages”*, while another dismisses them as “*minor quibbles*.” 

*(4)  The Consultation Model*. A consultation model of service delivery was the fourth theme to emerge from the qualitative phase of research. Though this theme was not selected by survey respondents as an important factor in their decision to work with TeleLink, 92% strongly agreed or agreed with the statement “*I am comfortable with the consultation model of service delivery*” and 56% identified “*I am only given cases that match my area of expertise*” as important or very important to their participation. Also, 80% identified telepsychiatry as a valuable resource to communities with limited access to specialized mental health care. In the interviews, psychiatrists indicate that the consultation model of service delivery encourages participation since the consulting psychiatrist does not accept ongoing responsibility for primary care, nor does he or she prescribe medication or do treatment. With a full-time job and regular patient load elsewhere, interview participants appreciate the opportunity to expand their practice without having to take on new patients. The financial incentives are attractive, as is the possibility to diversify their income sources. 

The majority of interview participants indicate that conducting psychiatric assessments via televideo does not significantly differ from those conducted face-to-face. Forty percent of survey respondents agreed or strongly agreed with this. One experienced psychiatrist, new to telepsychiatry, however, expresses a preference for face-to-face encounters and the need for more than one consultation session to do a thorough assessment. An added concern was the lack of feedback from rural clinicians regarding the effectiveness of his recommendations, stating “*I have no idea whether any of this is making any difference in kids' lives because I have not seen any follow-up data *…*. I have never seen a kid more than once.”*

Other concerns emerging from the qualitative data include the lack of information regarding resources in rural communities, rural clinicians who may be inexperienced due to high turnover rates, and having to “*tread carefully*” so as not be seen as a “*big shot coming in telling us *[*rural clinicians*]* what to do.*” There was also a lack of knowledge regarding liability and safety issues, clinical follow-up procedures, and funding mechanisms, with one consultant stating “*What is the renumeration*? *I do not know how I get paid; I do not know what it is. It is not all that transparent for some people.*” Many of these themes echo concerns expressed by survey respondents, as reported earlier. Despite these concerns, study participants acknowledge that, by providing timely, equitable access to specialist services, the TeleLink Program fills an important gap in the children's mental health system, “*distributing *(*psychiatric expertise*)* to a wide area*.”

## 5. Discussion

The delivery of psychiatric services using videoconferencing technology is an efficient, cost-effective [13–16], and user-friendly [17] approach that has been recommended for expansion in jurisdictions across Canada [7]. However, it has been noted that the growth and advancement of telepsychiatry programs may be constrained by human resource factors [7], including psychiatry workforce shortages [2, 18] and resistance from specialists to participate in such programs [19, 20]. As such, it is important to understand what factors may encourage or deter specialists from engaging with telepsychiatry programs. The TeleLink Mental Health Program offers a compelling case as it has regular access to approximately 40 child psychiatrists and, since its inception in 2000, retained much of its original workforce. The main factors contributing to this success as identified by study participants provide important strategies for the field in attracting and retaining child psychiatrists to a telepsychiatry program. 

For both survey and interview participants, organizational factors were identified as crucial to their participation in the TeleLink Program. Features such as efficient administrative staff, convenient setup, flexible work hours, and financial compensation allowed consulting psychiatrists to deliver services with minimal workload. In the literature, time, money [19], and technical skills [21] are identified as barriers to the uptake of telemedicine. This study and others [22, 23] indicate that such barriers can be significantly reduced by providing dedicated administrative and technological support. Also, adequate funding is critical, with 80% of survey participants identifying financial compensation as important or very important to their participation. For consultants working primarily in locations at a distance from the host site, costs associated with commuting could be mitigated by offering multiple consultations per visit, or offsite service provision, where feasible. 

A second set of factors linked to telepsychiatry participation is related to the technology itself. The majority of survey and interview participants reported being comfortable with the equipment and technology, as well as conducting assessments via televideo. However, results suggest that more could be done to improve technical aspects and provider comfort. As reported in a recent literature review [24], problems related to nonverbal communication and audiovisual quality are seen as drawbacks to telepsychiatry and that more needs to be done to improve infrastructure issues [19]. Also, training, ongoing technical support [25], and more user-friendly technology [26] have been shown to facilitate acceptance. Comfort levels could also be improved through better communication and information exchange, for example, providing consultants with information regarding available resources in local communities as well as postconsultation feedback regarding the appropriateness and usefulness of their recommendations. Finally, providing potential recruits and new consultants with an orientation package would help promote the program and provide answers to common questions, such as remuneration schemes and security issues.

A third factor in attracting consulting psychiatrists is active participation in postgraduate training; since 2005, all psychiatry residents at the University of Toronto are required to participate in at least two telepsychiatry consultations with the TeleLink Program. Additionally, three- and six-month electives are offered to interested residents [6]. This strategy is supported by survey respondents who believe that an organized effort is needed to introduce medical students and residents to specialized pediatric training early in their training, with 68% recommending that the University's curriculum committee make telepsychiatry mandatory for subspecialty training. In focus group data, psychiatry residents indicated that the opportunity to actively participate in televideo consultations was exciting and strengthened their interest in this model of psychiatric work. These data align with research that suggests exposure to positive training opportunities, and participation in psychiatry electives may enhance recruitment into psychiatry [27]. Given the downward trend in psychiatric recruitment [28–30], it behooves telepsychiatry programs to work with medical schools and psychiatric recruitment officers. To this end, the Canadian Mental Health Commission has encouraged the inclusion of telemental health instruction in medical schools [7]. 

## 6. Implications

This study aimed to gather objective data regarding how a telepsychiatry program in Toronto, Canada, is able to attract and keep child psychiatric consultants to the program, in the context of declining psychiatric recruitment and the difficulty in attracting psychiatrists to do this work, as indicated informally by similar programs in Canada and elsewhere. These results help us understand what aspects of telepsychiatry are most likely to attract and retain psychiatry practitioners, as well as aspects most appreciated by consulting psychiatrists and residents. Also, this study helps inform the effective recruitment of new graduates to the field, a current challenge for the TeleLink Program. In response to this challenge, the University of Toronto's Department of Psychiatry has made adult telepsychiatry a key goal and has asked the TeleLink Program to assist in setting up the program and attracting consulting psychiatrists. The results of this study will assist with this endeavour. 

More research is needed to better understand residents' experiences of participating in the TeleLink Program, and more specifically what might attract them to continue to work in telepsychiatry after graduation. Future research would include a multicentered study involving other telepsychiatry programs in Ontario and across Canada. 

## 7. Conclusion

This study offers important insights into addressing possible barriers in attracting psychiatrists and residents to a telepsychiatry program. It also provides an opportunity to think about wider recruitment and retention issues in the field of psychiatry. Understanding what aspects of the TeleLink Mental Health Program are most appreciated by consulting psychiatrists and what attracts residents to the Program offers useful strategies to telepsychiatry administrators and medical school educators seeking to attract, train, and retain psychiatry practitioners.

## Figures and Tables

**Table 1:** Frequency of assessments (*n* = 26).

How often do you provide assessments to the TeleLink Program?	Percentage
Never	23.1%
Rarely (less than 6 times per year)	19.2%
Occasionally (6–12 times per year)	23.1%
Frequently (more than 12 times per year)	34.6%

**Table 2:** Comfort with conducting assessments via televideo (*n* = 25).

How comfortable are you conducting assessments via televideo?	Percentage
Not comfortable (1–4)	20%
Moderately comfortable (5–7)	20%
Very comfortable (8–10)	60%

**Table 3:** Comfort with video equipment and technology (*n* = 25).

How comfortable are you with the video equipment and technology?	Percentage
Not comfortable (1–4)	4%
Moderately comfortable (5–7)	40%
Very comfortable (8–10)	56%

**Table 4:** Decisions to provide telepsychiatry services-importance rankings (*n* = 25).

How important are the following in your decision regarding whether or not to provide services to the TeleLink Program?						
	Very important	Important	Neutral	Somewhat important	Not at all important	Not applicable
Flexibility regarding number of hours I provide to the program	40% 10	32% 8	8% 2	8% 2	8% 2	4% 1
Given only cases that match my area of expertise	24% 6	36% 9	20% 5	16% 4	0% 0	4% 1
Support provided by telepsychiatry staff	36% 9	60% 15	0% 0	0% 0	0% 0	4% 1
Comfortable environment	24% 6	52% 13	20% 5	0% 0	0% 0	4% 1
Convenience	32% 8	44% 11	16% 4	4% 1	0% 0	4% 1
Financial incentives	20%	60%	12%	0%	0%	8%
	5	15	3	0	0	2
Opportunity to be part of larger group of professionals	8% 2	36% 9	36% 9	12% 3	0% 0	8% 2
Enhance capacity of local providers	32% 8	52% 13	8% 2	4% 1	0% 0	4% 1
Privileges associated with a hospital appointment	4% 1	8% 2	32% 8	4% 1	32% 8	20% 5
Novelty of the medium/technology	4% 1	8% 2	36% 9	8% 2	36% 9	8% 2
Maintaining ongoing relationship with rural agency	24% 6	36% 9	24% 6	0% 0	8% 2	8% 2
Providing service to underserved areas	52% 13	44% 11	0% 0	4% 1	0% 0	0% 0

**Table 5:** Psychiatric services via televideo (*n* = 25).

To what extent do you agree or disagree with the following statements regarding the provision of psychiatric services via televideo?						
	Strongly disagree	Disagree	Neutral	Agree	Strongly agree	Not applicable
Technology is sufficiently secure and confidential	0% 0	12% 3	12% 3	36% 9	40% 10	0% 0
I am concerned about safety & liability issues	8.3% 2	37.5% 9	20.8% 5	20.8% 5	12.5% 3	0% 0
Having others in room with patient is helpful in conducting an assessment	0% 0	0% 0	16% 4	48% 12	36% 9	0% 0
It is difficult to build rapport with others via televideo	8% 2	32% 8	24% 6	32% 8	4% 1	0% 0
I lack knowledge of the culture of rural communities to provide effective assistance	16% 4	48% 12	16% 4	20% 5	0% 0	0% 0
Information received prior to consultation is sufficient to conduct a proper assessment	0%	4%	32%	52%	8%	4%
	0	1	8	13	2	1
I have insufficient knowledge regarding the resources available in rural communities	4% 1	24% 6	20% 5	44% 11	8% 2	0% 0
Need more feedback from rural agencies about helpfulness of my recommendations	0% 0	8% 2	28% 7	40% 10	16% 4	8% 2

**Table 6:** Qualitative semistructured interview questions.

How often do you provide assessments to the TeleLink Program?	
How did you become involved in the TeleLink Program?	
What aspects of the program's organization/service delivery model facilitate and/or deter participation by psychiatrists and/or psychiatric residents?	
What are your thoughts regarding the provision of psychiatric services to rural and remote communities via televideo? How does that influence your decision to participate in the Program?	
How comfortable are you conducting assessments via televideo? How comfortable are you with the video equipment and technology?	
What circumstances or changes to the TeleLink Program would encourage you to provide some or more hours?	
If consulting to the TeleLink Program, what circumstances or changes would lead you to offer fewer hours?	
